# The chicken or the egg: low testosterone predisposes for COVID-19 or COVID-19 induces a decrease in testosterone?

**DOI:** 10.1186/s13054-021-03664-9

**Published:** 2021-07-07

**Authors:** Roeland F. Stolk, Henk J. van Leeuwen, Matthijs Kox, Marcel van Borren, Hans de Boer, Peter Pickkers

**Affiliations:** 1 Department of Intensive Care Medicine, Radboud University Medical Center, Nijmegen, The Netherlands; 2 Radboud Centre for Infectious Diseases, Radboud University Medical Center, Nijmegen, The Netherlands; 3 Department of Intensive Care Medicine, Hospital Rijnstate, Arnhem, The Netherlands; 4 Department of Laboratory Medicine, Hospital Rijnstate, Arnhem, The Netherlands; 5 Department of Internal Medicine, Hospital Rijnstate, Arnhem, The Netherlands

Recently, an inverse association between testosterone concentrations and disease severity in male but not female COVID-19 patients was reported [1]. COVID-19 is characterized by systemic inflammation and men are more likely to be severely affected than women. However, it is unclear whether lower testosterone levels predispose men towards a severe course in COVID-19, or that more severe COVID-19 infections induce a stronger decrease in circulating testosterone concentrations. Causality is difficult to establish in observational studies, as premorbid testosterone levels are not available and testosterone levels may already be reduced at hospital presentation. We investigated whether systemic inflammation lowers testosterone levels in men in vivo.

Following written informed consent, 6 healthy male volunteers (median [interquartile range] age of 24 [21 − 25] years and BMI of 29.1 [20.0–31.5] kg/m^2^) received a bolus of 1 ng/kg *E. Coli*-derived endotoxin, lipopolysaccharide (LPS). Detailed study procedures are described elsewhere [2]. Circulating concentrations of testosterone, luteinizing hormone (LH), follicle-stimulating hormone (FSH) and estradiol were measured in lithium heparin plasma using liquid chromatography-tandem mass spectrometry. Plasma cytokine levels were measured in ethylenediaminetetraacetic acid plasma using a Luminex assay (Milliplex, Millipore).

Experimental human endotoxemia induced systemic inflammation illustrated by increased plasma concentrations of several cytokines (Fig. 1A). Baseline testosterone levels were within reference for all subjects (median [IQR] 22.4 [18.8–26.5] nM) and decreased significantly following LPS administration (Fig. 1B), most prominently after 6 h (median [IQR] 14.0 [8.3–20.4] nM, − 37%). Interestingly, LH, FSH and estradiol levels were not affected by endotoxemia-induced inflammation (Fig. 1B, C). Levels of the adrenal glucocorticoid cortisol were strongly increased after LPS administration peaking after 2 h (median [IQR] 463 [354–563] nM, + 75%), Fig. 1B).

**Fig. 1:**
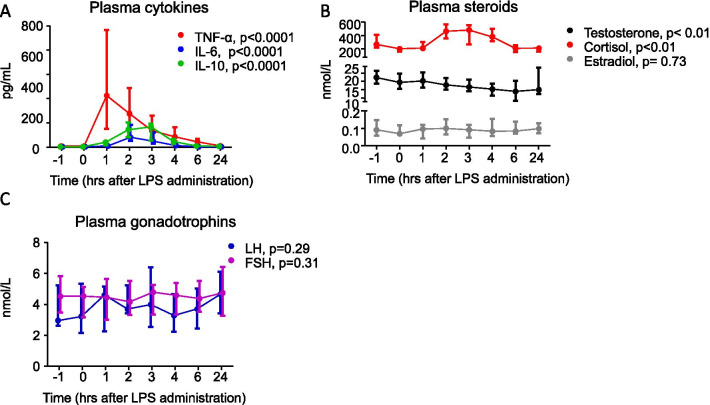
Cytokine, steroid and gonadotropin levels over time during human endotoxemia. Levels of circulating TNF-α, IL-6 and IL-10 (**A**), cortisol, testosterone and estradiol (**B**) and LH and FSH (**C**) over time, during human endotoxemia, after LPS administration in healthy volunteers. Data are presented as medians and IQR. LPS-induced changes over time were analyzed using the Friedman test) for all timepoints. *IL* interleukin, *LH* luteinizing hormone, *FSH* follicle stimulating hormone,

Within hours, systemic inflammation in healthy men is associated with a decrease in testosterone levels. This observation suggests that the low testosterone levels observed in COVID-19 patients are the result of inflammatory processes rather than a predisposing factor. The absence of an effect of endotoxemia on estradiol levels suggests that estradiol may be less sensitive to inflammation induced by a low dosage of LPS in our study.

It remains unclear whether the decrease in testosterone levels is an adaptive or maladaptive response in COVID-19. Although clinical trials into hormonal interventions in COVID-19 patients are already ongoing (e.g. the HITCH trial (clinicaltrials.gov identifier NCT04397718), it is unclear at this point whether interventions should be aimed at a further reduction or a supplementation of testosterone in men suffering from COVID-19.

## Data Availability

Individual data will be provided upon reasonable request.

## References

[1] Dhindsa S, Zhang N, McPhaul MJ, Wu Z, Ghoshal AK, Erlich EC (2021). Association of circulating sex hormones with inflammation and disease severity in patients with COVID-19. JAMA Netw Open.

[2] van Lier D, Geven C, Leijte GP, Pickkers P (2019). Experimental human endotoxemia as a model of systemic inflammation. Biochimie.

